# The Translation Process of Pharmaceutical Care Network Europe v9.00 to Bahasa Indonesia: An Instrument to Detect Drug-Related Problem

**DOI:** 10.21315/mjms2022.29.3.13

**Published:** 2022-06-28

**Authors:** Muhammad Aldila Satria, Retnosari Andrajati, Sudibyo Supardi

**Affiliations:** 1 Faculty of Pharmacy, Universitas Indonesia, Depok, West Java, Indonesia; 2 National Institute of Health Research and Development, Indonesian Ministry of Health, Jakarta, Indonesia

**Keywords:** drug-related problem, Pharmaceutical Care Network Europe, reliability, translation, validity

## Abstract

**Background:**

Drug-related problems (DRPs) remain a major health challenge in tertiary health services such as hospitals in Indonesia. These problems are detected and solved using classification systems such as Pharmaceutical Care Network Europe (PCNE). Therefore, this study aims to obtain a valid and reliable Bahasa Indonesia version of the PCNE.

**Methods:**

A draft of the Bahasa Indonesia version of the PCNE v9.00 was discussed by four experts from May to August 2020 using the Delphi method. Furthermore, the instrument was assessed for its readability, clarity and comprehensiveness by 46 hospital pharmacists throughout Indonesia. In October 2020, two pharmacists from Haji General Hospital, Makassar, Indonesia carried out the inter-rater agreement to assess 20 cases where the proportion of coding matches between both raters were observed.

**Results:**

The instrument was found to be valid after passing the face and content validity, and the Scale Content Validity Index (S-CVI) value for each PCNE domain was 0.91, 0.89, 0.93, 0.97 and 0.93, respectively. Moreover, there was a fair agreement between the two raters that ranged between 40%–90%. Also, kappa statistics showed a substantial agreement on the ‘Problems’ and ‘Causes’ domains.

**Conclusion:**

The Bahasa Indonesia version of the PCNE v9.00 instrument passed face and content validity as well as inter-agreement to be used in hospital settings.

## Introduction

Drug-related problems (DRP) are events or circumstances involving drug therapy that occur or potentially interfere with the achievement of desired health outcomes (1). Some of the factors that contribute to the emergence of DRP in patients include inappropriate prescription, ineffective treatment, underdose, non-compliance, etc (2). In Indonesia, DRP occurred in several chronic diseases such as diabetes (3), kidney (4) and heart failure (5). Therefore, there is an urgent need for understanding the importance of the pharmacist’s role in identifying, solving and reducing the incidence of DRP in patients (5).

Moreover, the documentation and classification of DRP can help pharmacists to identify and resolve DRP in a patient. Several classification systems, such as APS-Doc, Cipolle, DOCUMENT, Consensus of Granada, Strand Classification and the Pharmaceutical Care Network Europe (PCNE) system, are applied (6–7).

A DRP classification system needs to have an open hierarchical structure with clear definitions for each category described in the instrument to reduce ambiguity or multiple interpretations when carrying out the coding process (8). Furthermore, the ease of use is also a specific requirement of the DRP classification system, which must be acceptable. Therefore, the DRP classification system needs to be validated before it is widely used (8–9).

Besides having a good validity, an instrument also needs to have a good level of inter-rater reliability, which is a measure of the degree of agreement between two or more raters (10). This is required to determine the extent to which the raters consistently assign a precise value to each rated item (11). It is essential because the raters need to give the same value in the same conditions and cases.

In this study, the PCNE instrument was selected as the starting point because it is structured, detailed and also identifies the patient’s DRP status based on their problems, causes and interventions. Furthermore, the instrument has several translations including Spanish, Turkish, Croatian, French (12), Slovenia (7) and German (13). This study, therefore, aims to obtain, validate and determine the inter-rater agreement (percentage agreement and kappa statistics) of Bahasa Indonesia version of the PCNE version 9.00.

## Methods

### PCNE Instrument Usage Permit

There was difficulty in downloading the PCNE instrument from their official website; therefore, permission to translate the instrument into Bahasa Indonesia was requested from the PCNE organisation in the Netherlands. The instrument was then downloaded via the PCNE website. Version 9.00 of the PCNE instrument served as the starting point for the translation and it consisted of five main domains (problem, causes, planned intervention, intervention acceptance, and status of DRP), 24 primary domains and 84 secondary domains (14).

### Forward Translation

The study began with a forward translation of PCNE draft version 9.00 (English version) into Bahasa Indonesia by two independent sworn translators and the results were separately discussed. Furthermore, the translations were combined by paying attention to the excellent choice of words and pharmaceutical terms.

### Backward Translation

The instrument was again re-translated from Bahasa Indonesia to English by other translators. Similarly, the results were separately discussed.

### Face Validity

The combined draft was then given to four experts, including hospital pharmacists and academicians with master and doctoral qualifications. This assisted in the critical review of the translation results and suggestions for improvements to make the instrument easier to use. The process to reach consensus among experts was carried out using the Delphi method (15), which ensures that the expert panels do not know each other and report only to the researcher.

A draft of the Bahasa Indonesia version of the PCNE was sent to the expert panels in parallel and they were given time to provide a critical review of the translation. All the critical reviews from each expert panel were then combined and the instrument was refined. The draft was then returned to each expert panel and the process was repeated until they reached a consensus.

### Content Validity

This process involved a minimum of 20 clinical pharmacists who work in the hospital as respondents. They were asked to rate the criteria of readability, clarity and comprehensiveness of the instrument using a 5-point Likert scale. Furthermore, the Item Content Validity Index (I-CVI) and the Scale Content Validity Index (S-CVI) were calculated. The I-CVI compares the number of respondents that gave ratings of 3 and above with the total number of respondents. In contrast, the S-CVI is the average of the I-CVI values (16). The following formulae were used to calculate the I-CVI and S-CVI:


$$\begin{array}{l} \text{I-CVI}=\frac{\begin{array}{c} \text{Number of respondents who give ratings}\\ \text{more than or equals to }3\;\text{on Likert scale} \end{array}}{\text{Total respondents}}\\ \text{S-CVI}=\sum \frac{\text{I-CVI}}{\text{N}} \end{array}$$

where

N = number of valued I-CVI’s

The cutoff value for I-CVI and S-CVI are set based on Shrotryia and Dhanda, which is ≥ 0.78 for I-CVI and ≥ 0.8 for S-CVI (17).

### Inter-Rater Agreement

The inter-rater agreement involved two pharmacists from the Haji Regional General Hospital, Makassar, who conducted a DRP assessment on 20 selected patient cases using validated instruments. These cases were taken from patient medical records using consecutive sampling methods, which met the following eligibility criteria:

- Has undergone inpatient care at the hospital (recovered or died)
- Patients aged ≥ 18 years old
- Medical records are well documented

Before the test, the two raters were trained separately using five practice cases to familiarise them with the instrument. They were asked to provide a code in the ‘Problem’ and ‘Cause’ domains following the given patient’s DRP case using the Bahasa Indonesia version of PCNE. The coding consistency and chance agreement between the two raters were determined by calculating percentage agreement and kappa statistics. The percentage agreement is the ratio of the number of cases in which both raters gave the same code to the total number of cases. The formula below is used to calculate percentage agreement (18):


$$\text{Percentage agreement}=\frac{\text{Number of concordant cases}}{\text{Total number of cases}}\times 100\;\%$$

The kappa statistics were carried out using IBM SPSS^®^ version 24 software and its interpretation is showed in Table 1 (18).

## Results

The PCNE classification version 9.00 was translated into Bahasa Indonesia by two sworn translators that did not meet. Furthermore, a reconciliation process was conducted with each translator regarding the translation results, which were then combined. The draft of the translated instrument as shown in Appendix 1 were submitted to the experts for a critical review. After two sessions of discussion with the expert panels, the following changes were incorporated:

- Addition of the conjunctions in the *Penerimaan Intervensi* and *Status MTO* domain. For example, the sentence *Intervensi tidak diterima: tidak layak* in domain A2.1 was changed to *Intervensi tidak diterima karena tidak dapat dilakukan*.
- Changes were made in the word structure of the subdomain to make the sentences easier to understand for the users. For example, the meaning of sentences, such as *Bentuk obat yang tidak sesuai (untuk pasien ini)* in domain C2.1 was changed to *Bentuk sediaan obat yang tidak sesuai dengan pasien*.
- Sentences were simplified to enable a more concise reading. For example, the sentence *Pada kasus tertentu ada efek samping obat merugikan yang (mungkin) terjadi* in domain P2.1 was changed to *Kejadian obat yang merugikan (mungkin) terjadi*.

**Figure 1:**
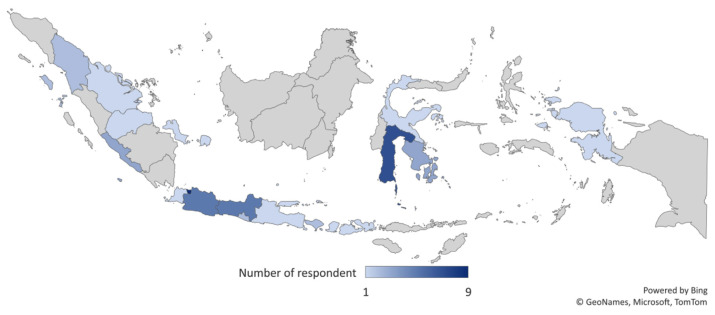
Distribution of the content validity respondents throughout Indonesia

A total of 46 hospital pharmacists (Table 2) were recruited from 17 provinces throughout Indonesia (Figure 1) in content validity. The majority of respondents filled 3 and 4 on a 5-point Likert scale, followed by 5, and a few filled 1 and 2. Moreover, the respondents assessed the instrument using four aspects including readability, clarity, ambiguity and comprehensiveness of the instrument. Also, the I-CVI and S-CVI values of each domain’s instrument ranged between 0.85–0.98 and 0.89–0.97, respectively (Table 3). The final version of PCNE after conducting the face and content validity is the Indonesia version shown in Appendix 2.

In addition, the inter-rater agreement involved two pharmacists that worked at Haji General Regional Hospital, Makassar, as raters. The first had 15 years of working experience in the hospital, while the second had 8 years. However, they are not familiar with PCNE instruments, therefore, they were trained using five practice cases.

After assessing the DRP cases of 20 patients, the percentage agreement was 90% higher in the ‘problems’ domain for both the primary and secondary domains, respectively. While in the ‘causes’ domain, it was much lower by 60% and 40% on the primary and secondary domains, respectively. Furthermore, kappa statistics were performed to calculate the chance agreement of two raters when identifying the DRP on a case using PCNE. The result showed a significant agreement between the two raters on ‘problems’ domain (κ = 0.615 [95% CI: 0.149, 1.081]; *P* = 0.003) and ‘Causes’ domain (κ = 0.612 [95% CI: 0.298, 0.910]; *P* = 0.003).

## Discussion

This study is the first to translate, validate and determine the inter-rater agreement of the translated PCNE into Bahasa Indonesia. After the forward (English-Bahasa Indonesia) and backward (Bahasa Indonesia-English) translations, some differences were noticed. These include the changes in word structure, especially in the ‘intervention acceptance’ domain, which is different from the original version because that of Bahasa Indonesia uses conjunctions to make the domain easier for users to understand. According to the suggestions from experts and respondents during the validation process, the changes in the number of word structure compared to the original version before the translation were influenced by the changes in the word structure in Bahasa Indonesia. However, the translation does not differ significantly in the interpretation of the main point of the sentence.

Furthermore, the I-CVI and S-CVI values have a high content validity level because they passed Shrotryia and Dhanda’s content validity levels of ≥ 0.78 and 0.8, respectively. However, this value is different from the value reported by Koubaity et al. (12) on the validation of PCNE French. Also, the values of I-CVI and S-CVI were in the range of 0.9–1.0 versus 0.85–0.97 in previous studies.

There is also a high consistency in the ‘problems’ domain of the instrument on an inter-rater agreement study. However, the ‘causes’ domain has low consistency, which differs from the results of Koubaity et al. in 2019 and Schindler et al. in 2020 (12–13). Furthermore, these two studies yielded a percentage agreement between 59%–100% and 57.4%–77.3%, respectively. Several factors resulted in the low consistency between the two raters of the ‘causes’ domain. First, this study used a small sample of pharmacists compared to Schindler et al. (13) which considered a total of 32 pharmacists. Second, the variety of codes and the ability to code the case summaries led to different perspectives between the two raters, causing the domain to have low consistency (12). Finally, the raters admitted that it was quite challenging to choose the correct code for a patient’s case, especially in the ‘causes’ domain. Moreover, the two previous studies reported that the raters had difficulty determining the correct code for a given case (13).

The kappa statistics showed a high degree of agreement on both ‘problem’ and ‘causes’ domains. The value was higher compared to others such as DOCUMENT (0.53 versus 0.615) (19) and GSASA V2 (0.52 versus 0.615) (20), lower than APS-Doc (0.68 versus 0.615) (21) and similar with the classification developed by the Pharmaceutical Society of Singapore (ILTC DRP Classification System) (0.614 versus 0.615) (8). Furthermore, it is believed that the low value of kappa in this study is because the raters are not too familiar with the instrument. Therefore, using the instrument frequently may increase the value of kappa. This is influenced by the raters’ level of knowledge and experience.

This study has certain limitations. First, the instrument does not assess the inter-rater agreement on the ‘planned intervention,’ ‘intervention acceptance,’ and ‘status of DRP’ domains because only secondary data were used. Second, the inter-rater agreement is still limited to only two assessors due to the unfamiliarity of this instrument in daily pharmacy practices in Indonesian hospitals. Furthermore, construct validity, such as convergence to see the instrument’s reliability under different conditions (22), was not performed. Therefore, further studies are suggested to focus mainly on reliability testing by involving more pharmacists and performing the construct validity.

## Conclusion

The PCNE v9.00, Bahasa Indonesia version has passed content validity and inter-agreement for use in pharmacy practice in both hospitals and academic settings. Further study is suggested to focus mainly on inter-rater reliability tests using more pharmacists to measure the validity of the instruments in various conditions in hospital settings.

## Figures and Tables

**Table 1:** Interpretation of kappa values

Kappa values	Interpretation
< 0.00	Poor
0.00–0.20	Slight
0.21–0.40	Fair
0.41–0.60	Moderate
0.61–0.80	Substantial
0.81–1.00	Almost perfect

Note: Source: (10)

**Table 2:** Demographics of respondents who participates in content validity

Variables	*n* (%)	Total (%)
Gender		46 (100)
Men	17 (37.0)	
Women	29 (63.0)	
Education		
Profession	38 (82.6)	46 (100)
Master	8 (17.4)	
Work experience		
0–5 years	26 (56.5)	
5–10 years	9 (19.5)	46 (100)
> 10 years	11 (24.0)	

**Table 3:** I-CVI and S-CVI value on validity content

Aspect	I-CVI^a^ on the domain (*n* = 46)				

	Problems	Causes	Planned intervention	Intervention acceptance	Status of DRP
Words/sentences in this domain are readable	0.96	0.93	0.96	0.96	0.96
Words/sentences in this domain are straightforward and easy to understand	0.87	0.87	0.91	0.98	0.93
I do not see any ambiguous words/sentences in this domain	0.87	0.85	0.91	0.96	0.91
The description of each domain is well defined	0.91	0.89	0.93	0.98	0.91
S-CVI^b^	0.90	0.89	0.93	0.97	0.93

Notes:

a I-CVI = Item Content Validity Index;

b S-CVI = Scale Content Validity Index

## Appendix 1 Bahasa Indonesia version of Pharmaceutical Care Network Europe (PCNE) (after translation)

### *Pharmaceutical Care Network Europe v9.00* versi Indonesia

**Table:**

	Kode	Domain Primer	Domain Sekunder
Masalah	P1	**Efektivitas pengobatan** Terdapat (potensial) masalah dengan (kurangnya) efek farmakoterapi	P1.1 Tidak ada efek dari pengobatan P1.2 Pengaruh terapi obat tidak optimal P1.3 Gejala atau indikasi yang tidak diobati
	P2	**Keamanan pengobatan** Pasien menderita, atau bisa menderita, akibat suatu kejadian obat yang merugikan	P2.1 Pada kasus tertentu ada efek samping obat merugikan yang (mungkin) terjadi
	P3	Lainnya	P3.1 Masalah dengan efektivitas biaya pengobatan P3.2 Pengobatan yang tidak perlu P3.3 Masalah / keluhan yang tidak jelas. Diperlukan klarifikasi lebih lanjut (harap gunakan hanya sebagai alternatif)
Penyebab	C1	**Pemilihan obat** Penyebab DRP terkait dengan pemilihan obat	C1.1 Obat yang tidak sesuai dengan pedoman / formularium C1.2 Obat yang tidak sesuai (sesuai pedoman tetapi dinyatakan bertentangan) C1.3 Tidak ada indikasi untuk obat C1.4 Kombinasi obat-obatan yang tidak tepat, atau obat-obatan dan herbal, atau obat-obatan dan suplemen makanan C1.5 Duplikasi yang tidak tepat dari kelompok terapeutik atau bahan aktif C1.6 Pengobatan yang tidak ada atau tidak lengkap terlepas dari indikasi yang ada C1.7 Terlalu banyak obat yang diresepkan untuk indikasi
	C2	**Bentuk obat** Penyebab DRP dapat terkait dengan pemilihan bentuk obat	C2.1 Bentuk obat yang tidak sesuai (untuk pasien ini)
	C3	**Pemilihan dosis** Penyebab DRP dapat terkait dengan pemilihan jadwal dosis	C3.1 Dosis obat terlalu rendah C3.2 Dosis obat terlalu tinggi C3.3 Regimen dosis tidak cukup sering C3.4 Regimen dosis terlalu sering C3.5 Instruksi waktu dosis salah, tidak jelas atau hilang
	C4	Durasi pengobatan Penyebab DRP dapat terkait dengan durasi pengobatan	C4.1 Durasi pengobatan terlalu singkat C4.2 Durasi pengobatan terlalu lama
	C5	**Penyiapan obat** Penyebab DRP dapat terkait dengan logistik proses peresepan dan penyiapan obat	C5.1 Obat yang diresepkan tidak tersedia C5.2 Informasi yang diperlukan tidak tersedia C5.3 Obat yang salah, kekuatan sediaan atau dosis yang disarankan (OTC) C5.4 Obat atau kekuatan sediaan yang salah disiapkan
	C6	**Proses penggunaan obat** Penyebab DRP dapat terkait dengan cara pasien mendapatkan obat yang diberikan oleh tenaga kesehatan atau pengasuh, terlepas dari instruksi yang tepat (pada label)	C6.1 Waktu pemberian atau interval pemberian dosis yang tidak tepat C6.2 Obat yang diberikan kurang C6.3 Obat berlebihan C6.4 Obat tidak diberikan sama sekali C6.5 Obat yang diberikan salah C6.6 Obat diberikan melalui rute yang salah
	C7	**Terkait pasien** Penyebab DRP dapat terkait dengan pasien dan perilakunya (sengaja atau tidak disengaja)	C7.1 Pasien menggunakan / mengambil obat yang lebih sedikit dari yang diresepkan atau tidak menggunakan obat sama sekali C7.2 Pasien menggunakan / mengambil lebih banyak obat daripada yang diresepkan C7.3 Pasien menyalahgunakan obat (penggunaan berlebihan yang tidak diatur) C7.4 Pasien menggunakan obat yang tidak perlu C7.5 Pasien memakan makanan yang berinteraksi C7.6 Pasien menyimpan obat secara tidak tepat C7.7 Waktu atau interval pemberian dosis yang tidak tepat C7.8 Pasien memberikan / menggunakan obat dengan cara yang salah C7.9 Pasien tidak dapat menggunakan obat / bentuk sesuai petunjuk C7.10 Pasien tidak dapat memahami instruksi dengan benar
	C8	**Terkait transfer pasien** Penyebab DRP dapat terkait dengan transfer pasien antara perawatan primer, sekunder, dan tersier, atau transfer dalam satu institusi perawatan.	C8.1 Tidak ada rekonsiliasi obat saat transfer pasien C8.2 Tidak ada daftar obat terbaru yang tersedia. C8.3 Informasi pengeluaran / transfer tentang obat-obatan tidak lengkap atau hilang C8.4 Informasi klinis yang tidak memadai tentang pasien. C8.5 Pasien belum menerima obat yang diperlukan saat pemulangan
	C9	Lainnya	C9.1 Tidak terdapat hasil pemantauan yang sesuai (termasuk TDM) C9.2 Penyebab lain; sebutkan C9.3 Tidak ada penyebab yang jelas
Intervensi Terencana	I0	**Tidak ada intervensi**	I0.1 Tanpa Intervensi
	I1	Pada tingkat penulis resep	I1.1 Penulis resep hanya menginformasikan I1.2 Penulis resep meminta informasi I1.3 Intervensi diusulkan kepada penulis resep I1.4 Intervensi dibahas dengan penulis resep
	I2	Pada tingkat pasien	I2.1 Konseling (obat) pasien I2.2 Informasi yang tersedia (hanya) tertulis I2.3 Pasien telah dirujuk ke dokter tersebut I2.4 Disampaikan kepada anggota keluarga / pengasuh
	I3	**Pada tingkat obat**	I3.1 Obat diubah menjadi … I3.2 Dosis diubah menjadi … I3.3 Formulasi diubah menjadi … I3.4 Petunjuk penggunaan diubah menjadi… I3.5 Obat ditunda atau dihentikan I3.6 Obat dimulai
	I4	**Lainnya**	I4.1 Intervensi lainnya (sebutkan) I4.2 Efek samping dilaporkan ke pihak berwenang
Penerimaan Intervensi	A1	**Intervensi diterima**	A1.1 Intervensi diterima dan diimplementasikan sepenuhnya A1.2 Intervensi diterima, dilaksanakan sebagian A1.3 Intervensi diterima tetapi tidak diterapkan A1.4 Intervensi diterima, implementasi tidak diketahui
	A2	**Intervensi tidak diterima**	A2.1 Intervensi tidak diterima: tidak layak A2.2 Intervensi tidak diterima: tidak ada kesepakatan A2.3 Intervensi tidak diterima: alasan lain (sebutkan) A2.4 Intervensi tidak diterima: alasan tidak diketahui
	A3	Lainnya	A3.1 Intervensi diusulkan, penerimaan tidak diketahui A3.2 Intervensi tidak diusulkan
Status DRP	O0	**Tidak diketahui**	O0.1 Status masalah tidak diketahui
	O1	**Terselesaikan**	O1.1 Masalah terselesaikan sepenuhnya
	O2	**Sebagian diselesaikan**	O2.1 Masalah diselesaikan sebagian
	O3	Tidak terselesaikan	O3.1 Masalah tidak terselesaikan, kurangnya kerjasama dengan pasien O3.2 Masalah tidak terselesaikan, kurangnya kerja sama dengan penulis resep O3.3 Masalah tidak terselesaikan, intervensi tidak efektif O3.4 Tidak perlu atau kemungkinan untuk menyelesaikan masalah

## Appendix 2 Bahasa Indonesia version of Pharmaceutical Care Network Europe (PCNE) (after validity test)

### *Pharmaceutical Care Network Europe v9.00* versi Indonesia

**Table:**

	Kode	Domain Primer	Domain Sekunder
Masalah	P1	**Efektivitas pengobatan** Terdapat masalah yang berpotensi mengurangi efek farmakoterapi	P1.1 Tidak ada efek dari terapi obat P1.2 Efek terapi obat tidak optimal P1.3 Gejala atau indikasi yang tidak diobati
	P2	**Keamanan pengobatan** Pasien mengalami, atau dapat mengalami efek obat yang merugikan	P2.1 Kejadian obat yang merugikan (mungkin) terjadi
	P3	Lainnya	P3.1 Masalah pengobatan yang berkaitan dengan efektivitas biaya P3.2 Pengobatan yang tidak diperlukan P3.3 Masalah terkait obat yang tidak jelas, sehingga memerlukan klarifikasi lebih lanjut (harap gunakan hanya sebagai alternatif)
Penyebab	C1	**Pemilihan obat** Masalah Terkait Obat (MTO) terjadi karena pemilihan obat	C1.1 Obat tidak sesuai dengan pedoman / formularium C1.2 Obat sesuai pedoman, namun terdapat kontraindikasi C1.3 Tidak ada indikasi untuk obat C1.4 Kombinasi tidak tepat misalnya obat-obat, obat-herbal, atau obat-suplemen C1.5 Duplikasi dari kelompok terapeutik atau bahan aktif yang tidak tepat C1.6 Pengobatan tidak diberikan atau tidak lengkap walaupun terdapat indikasi C1.7 Terlalu banyak obat yang diresepkan untuk satu indikasi
	C2	**Bentuk obat** Masalah Terkait Obat (MTO) terjadi karena pemilihan bentuk sediaan obat	C2.1 Bentuk sediaan obat yang tidak sesuai dengan pasien
	C3	**Pemilihan dosis** Masalah Terkait Obat (MTO) terjadi karena pemilihan dosis obat	C3.1 Dosis obat terlalu rendah C3.2 Dosis obat terlalu tinggi C3.3 Regimen dosis kurang C3.4 Regimen dosis terlalu sering C3.5 Instruksi waktu pemberian dosis salah, tidak jelas atau tidak ada
	C4	**Durasi pengobatan** Masalah Terkait Obat (MTO) terjadi karena durasi pengobatan	C4.1 Durasi pengobatan terlalu singkat C4.2 Durasi pengobatan terlalu lama
	C5	**Penyiapan obat** Masalah Terkait Obat (MTO) terjadi karena proses ketersediaan obat yang diresepkan dan proses penyiapannya	C5.1 Obat yang diresepkan tidak tersedia C5.2 Informasi yang diperlukan tidak tersedia C5.3 Salah obat, kekuatan sediaan atau regimen dosis yang disarankan (khusus OTC/obat bebas) C5.4 Salah penyiapan obat atau kekuatan dosis
	C6	**Proses penggunaan obat** Masalah Terkait Obat (MTO) terjadi karena penggunaan obat pasien terlepas dari instruksi yang tepat (pada label) oleh tenaga medis atau perawat	C6.1 Waktu pemberian obat atau interval dosis tidak tepat C6.2 Obat yang diberikan kurang C6.3 Obat yang diberikan berlebih C6.4 Obat tidak diberikan sama sekali C6.5 Obat yang diberikan salah C6.6 Obat diberikan melalui rute yang salah
	C7	**Terkait pasien** Masalah Terkait Obat (MTO) terjadi karena pasien dan perilakunya (sengaja atau tidak sengaja)	C7.1 Pasien menggunakan obat lebih sedikit dari yang diresepkan atau tidak menggunakan obat sama sekali C7.2 Pasien menggunakan obat lebih banyak dari yang diresepkan C7.3 Pasien menyalahgunakan obat (tidak sesuai anjuran) C7.4 Pasien menggunakan obat yang tidak perlu C7.5 Pasien mengonsumsi makanan yang menyebabkan interaksi obat C7.6 Pasien menyimpan obat secara tidak tepat C7.7 Waktu atau interval pemberian dosis yang tidak tepat C7.8 Pasien menggunakan obat dengan cara yang salah C7.9 Pasien tidak dapat menggunakan obat / bentuk sediaan sesuai petunjuk C7.10 Pasien tidak dapat memahami instruksi dengan benar
	C8	**Terkait transfer pasien** Masalah Terkait Obat (MTO) terkait dengan perpindahan pasien antara perawatan primer, sekunder, dan tersier, atau dalam satu ruang perawatan	C8.1 Tidak ada rekonsiliasi obat saat pasien dipindahkan C8.2 Tidak ada daftar obat terbaru yang tersedia. C8.3 Informasi tentang obat-obatan pada saat pemulangan/ transfer tidak lengkap atau hilang C8.4 Informasi klinis tentang pasien tidak memadai C8.5 Pasien belum menerima obat yang diperlukan saat pemulangan
	C9	Lainnya	C9.1 Tidak terdapat hasil pemantauan terapi obat yang sesuai (termasuk TDM/*Therapeutic Drug Monitoring*) C9.2 Penyebab lain; sebutkan....... C9.3 Tidak ada penyebab yang jelas
Rencana Intervensi	I0	**Tidak ada intervensi**	I0.1 Tanpa Intervensi
	I1	Pada tingkat dokter penulis resep	I1.1 Dokter penulis resep hanya diinformasikan I1.2 Dokter penulis resep meminta informasi I1.3 Intervensi diusulkan kepada dokter penulis resep I1.4 Intervensi dibahas dengan dokter penulis resep
	I2	Pada tingkat pasien	I2.1 Konseling kepada pasien terkait obat I2.2 Tersedia informasi tertulis I2.3 Pasien disarankan kembali ke dokter I2.4 Menyampaikan kepada anggota keluarga / pengasuh
	I3	**Pada tingkat obat**	I3.1 Obat diubah menjadi … I3.2 Dosis diubah menjadi … I3.3 Formulasi diubah menjadi … I3.4 Petunjuk penggunaan diubah menjadi… I3.5 Obat ditunda atau dihentikan I3.6 Obat dimulai
	I4	**Lainnya**	I4.1 Intervensi lainnya (sebutkan) I4.2 Efek samping dilaporkan ke pihak berwenang
Penerimaan Intervensi	A1	**Intervensi diterima**	A1.1 Intervensi diterima dan diimplementasikan sepenuhnya A1.2 Intervensi diterima namun hanya diimplementasikan sebagian A1.3 Intervensi diterima namun tidak diimplementasikan A1.4 Intervensi diterima namun implementasi tidak diketahui
	A2	Intervensi tidak diterima	A2.1 Intervensi tidak diterima karena tidak dapat dilakukan A2.2 Intervensi tidak diterima karena tidak disetujui A2.3 Intervensi tidak diterima karena alasan lain (sebutkan) A2.4 Intervensi tidak diterima karena alasan tidak diketahui
	A3	**Lainnya**	A3.1 Intervensi diusulkan namun penerimaan tidak diketahui A3.2 Intervensi tidak diusulkan
Status MTO	O0	**Tidak diketahui**	O0.1 Status masalah tidak diketahui
	O1	**Terselesaikan**	O1.1 Masalah terselesaikan sepenuhnya
	O2	**Sebagian diselesaikan**	O2.1 Masalah diselesaikan sebagian
	O3	Tidak terselesaikan	O3.1 Masalah tidak terselesaikan karena kurangnya kerjasama dengan pasien O3.2 Masalah tidak terselesaikan karena kurangnya kerja sama dengan penulis resep O3.3 Masalah tidak terselesaikan karena intervensi tidak efektif O3.4 Tidak perlu atau tidak memungkinkan untuk menyelesaikan masalah
